# The Differential Influence of Cold Ischemia Time on Outcome After Liver Transplantation for Different Indications—Who Is at Risk? A Collaborative Transplant Study Report

**DOI:** 10.3389/fimmu.2020.00892

**Published:** 2020-05-12

**Authors:** Vladimir J. Lozanovski, Bernd Döhler, Karl Heinz Weiss, Arianeb Mehrabi, Caner Süsal

**Affiliations:** ^1^Department of General, Visceral and Transplantation Surgery, University Hospital Heidelberg, Heidelberg, Germany; ^2^Liver Cancer Center Heidelberg (LCCH), University of Heidelberg, Heidelberg, Germany; ^3^Institute of Immunology, University Hospital Heidelberg, Heidelberg, Germany; ^4^Department of Internal Medicine IV, University Hospital Heidelberg, Heidelberg, Germany

**Keywords:** cold ischemia time, CIT, liver transplantation, extended donor criteria, EDC, collaborative transplant study, CTS, outcome

## Abstract

**Introduction:** Despite increasing awareness of the negative impact of cold ischemia time (CIT) in liver transplantation, its precise influence in different subgroups of liver transplant recipients has not been analyzed in detail. This study aimed to identify liver transplant recipients with an unfavorable outcome due to prolonged cold ischemia.

**Methods:** 40,288 adult liver transplantations, performed between 1998 and 2017 and reported to the Collaborative Transplant Study were analyzed.

**Results:** Prolonged CIT significantly reduced graft and patient survival only during the first post-transplant year. On average, each hour added to the cold ischemia was associated with a 3.4% increase in the risk of graft loss (hazard ratio (HR) 1.034, *P* < 0.001). The impact of CIT was strongest in patients with hepatitis C-related (HCV) cirrhosis with a 24% higher risk of graft loss already at 8–9 h (HR 1.24, 95% CI 1.05–1.47, *P* = 0.011) and 64% higher risk at ≥14 h (HR 1.64, 95% CI 1.30–2.09, *P* < 0.001). In contrast, patients with hepatocellular cancer (HCC) and alcoholic cirrhosis tolerated longer ischemia times up to <10 and <12 h, respectively, without significant impact on graft survival (*P* = 0.47 and 0.42). In HCC patients with model of end-stage liver disease scores (MELD) <20, graft survival was not significantly impaired in the cases of CIT up to 13 h.

**Conclusion:** The negative influence of CIT on liver transplant outcome depends on the underlying disease, patients with HCV-related cirrhosis being at the highest risk of graft loss due to prolonged cold ischemia. Grafts with longer cold preservation times should preferentially be allocated to recipients with alcoholic cirrhosis and HCC patients with MELD <20, in whom the effect of cold ischemia is less pronounced.

## Introduction

Liver transplantation improves the underlying liver dysfunction, involves radical oncological resection, and is the only promising treatment for patients with end-stage liver disease and patients with hepatocellular carcinoma (HCC) (1–3). Because of the chronic organ shortage in most countries and in Eurotransplant, less than optimal, extended donor criteria (EDC) grafts are used to expand the organ pool (2, 4). Cold ischemia time (CIT) is a factor that occurs during the allocation and it is considered a major extended donor criterion (maEDC) that affects graft and patient survival along with macrovesicular steatosis and donor age (2, 5). Cold ischemia increases the risk of graft failure and early HCC recurrence, and graft outcome depends on its ability to recover from the ischemia injury (2, 6, 7). Therefore, organs with prolonged cold ischemia are often discarded as unsuitable for transplantation (2). To address this problem, we suggested an allocation algorithm that balances the maEDC with the recipient's health condition, and considers maEDC grafts an acceptable alternative for transplant candidates with lower laboratory Model of End-Stage Liver Disease (labMELD) scores who generally are in a better condition (2). Based on data from the Collaborative Transplant Study (CTS), we reported recently that donor age had a differential influence on graft survival depending on the indication for liver transplantation (8). Transplant recipients with HCC were less affected by advanced donor age whereas donor age influenced outcome strongly in patients with hepatitis C (HCV)-related cirrhosis. However, the impact of prolonged cold ischemia in patients with different underlying diseases was not investigated. Although the awareness of the negative impact of CIT has generally increased, the information on CIT's influence on outcome of maEDC grafts is scarce. Moreover, the accepted limits for CIT are subject to regional differences (5, 8–10). This study aimed to identify liver transplant recipients whose grafts are less affected from a prolonged cold ischemia, and to describe risk factors associated with an adverse outcome following transplantation of such organs.

## Methods

### Study Population

All data were obtained from the CTS (www.ctstransplant.org). Since 1982, CTS collects data from solid-organ transplants worldwide on a voluntary base, continuously reports general information on transplantation outcomes and specific clinical issues, and takes into account the confidentiality of patients as well as transplant centers. The well-structured follow-up concept and the incorporation of available registry data guarantee a high level of data integrity (11).

We analyzed data from 40,288 deceased donor primary liver transplantations reported to CTS and performed from January 1st, 1998 to December 31st, 2017 in adult patients with alcoholic liver cirrhosis or cirrhosis due to HCV and HCC. Less frequent original diseases such as autoimmune disorders, cryptogenic cirrhosis, congenital diseases, hepatitis B, metabolic disorders, primary biliary cirrhosis, and primary sclerosing cholangitis were analyzed as a separate group. Patients with missing data on CIT, transplanted because of acute hepatic failure, recipients of organs from <18-year-old donors, split liver or multi-organ transplants were excluded. The MELD score was available to CTS after 2006.

Graft failure was defined as insufficient liver function to keep the patient alive, leading to death or re-transplantation, whereas patient survival was defined as the time between the primary transplantation and death or last known contact.

### Statistical Analysis

Statistical analysis was performed using IBM SPSS Statistics version 25.0 (SPSS Inc., IBM Corporation, Somers, NY, USA). Survival rates were analyzed using the Kaplan-Meier method with the Mantel Cox log rank test of trend. To avoid possible influences from demographic differences, multivariable Cox regression analysis was used to calculate the hazard ratio (HR) and 95% confidence intervals (95% CI). The following confounders were considered: geographical region (country or region), year of transplantation, recipient age and race, donor age and race, cause of donor death, recipient and donor gender combinations, general evaluation of the patient, original disease, donation after cardiac death, donor history of hypertension, immunosuppressive regimen, induction therapy, urgency, and CIT. A two-sided *P*-value of <0.05 was considered statistically significant.

## Results

We analyzed 40,288 primary adult liver transplantations from 109 centers in 24 countries. 10,953 patients had HCC, 9,569 were transplanted because of HCV-related liver cirrhosis, 7,878 had alcoholic cirrhosis, and in 11,888 patients the underlying disease included autoimmune disorders, cryptogenic cirrhosis, congenital diseases, hepatitis B virus, metabolic disorders, primary biliary cirrhosis or primary sclerosing cholangitis. Confounders were unevenly distributed between the most common underlying diseases; e.g., patients with HCC received notably more grafts from ≥65-year-old donors and the lowest number of grafts with CIT ≥10 h. Demographics and confounders are shown in Table 1.

**Table 1:** Demographics of study patients, *n* (%) or mean ± SD, *P* < 0.001 for all characteristics.

**Characteristic**	**Unknown (%)**	**Underlying disease**			
		**HCC**	**HCV-cirrhosis**	**Alcoholic cirrhosis**	**Other**
		***n* = 10,953**	***n* = 9,569**	***n* = 7,878**	***n* = 11,888**
Geographical region	–				
Europe		10,110 (92%)	8,285 (87%)	7,264 (92%)	10,283 (86%)
Other		843 (8%)	1,284 (13%)	614 (8%)	1,605 (14%)
Transplant year	–				
1998–2007		4,766 (44%)	5,751 (60%)	3,619 (46%)	6,467 (54%)
2008–2017		6,187 (56%)	3,818 (40%)	4,259 (54%)	5,421 (46%)
Recipient sex	–				
Female		1,853 (17%)	2,461 (26%)	1,604 (20%)	5,271 (45%)
Male		9,028 (83%)	6,993 (74%)	6,244 (80%)	6,483 (55%)
Recipient age (years)	–				
18–64		9,420 (86%)	8,951 (94%)	7,286 (92%)	10,860 (91%)
≥65		1,533 (14%)	618 (6%)	592 (8%)	1,028 (9%)
Mean ± SD		56.2 ± 8.1	52.6 ± 8.4	54.0 ± 7.8	49.2 ± 12.3
Donor age (years)	–				
18–64		7,328 (67%)	7,552 (79%)	5,938 (75%)	9,579 (81%)
≥65		3,625 (33%)	2,017 (21%)	1,940 (25%)	2,309 (19%)
Mean ± SD		55.0 ± 16.9	50.2 ± 16.3	51.8 ± 16.4	49.2 ± 16.5
Cold ischemia time (h)	–				
≤ 5		1,576 (14%)	1,341 (14%)	1,038 (13%)	1,628 (14%)
6–9		6,405 (58%)	5,098 (53%)	4,183 (53%)	6,220 (52%)
10–13		2,659 (24%)	2,645 (28%)	2,276 (29%)	3,507 (30%)
≥14		323 (3%)	485 (5%)	381 (5%)	533 (4%)
Mean ± SD		8.1 ± 2.7	8.5 ± 3.3	8.5 ± 2.9	8.4 ± 2.9
Cause of donor death	5.4				
CVA		6,796 (65%)	5,512 (62%)	4,708 (63%)	6,982 (63%)
Trauma		2,212 (21%)	2,098 (24%)	1,508 (20%)	2,500 (22%)
Other		1,441 (14%)	1,273 (14%)	1,267 (17%)	1,643 (15%)
Calcineurin inhibitors	38.7				
Cyclosporine		2,021 (26%)	1,942 (34%)	1,074 (24%)	1,889 (28%)
Tacrolimus		5,182 (66%)	3,341 (58%)	3,005 (68%)	4,307 (64%)
None		613 (8%)	486 (8%)	346 (8%)	489 (7%)

*HCC, hepatocellular carcinoma; HCV, hepatitis C virus; SD, standard deviation; CVA, cerebrovascular accident*.

**Figure 1:**
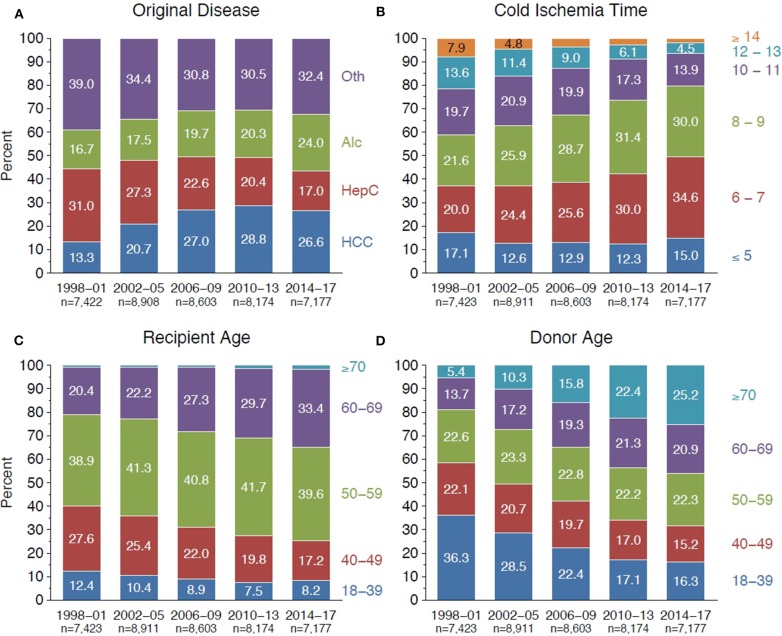
Development of **(A)** original underlying disease, **(B)** cold ischemia time (h), **(C)** recipient age (years), and **(D)** donor age (years) for first deceased donor liver transplants of adult recipients (*P* < 0.001 for all parameters).

During 1998–2001, chronic HCV infection was the leading cause of liver cirrhosis (31%), however, the proportion of recipients with HCV-related liver cirrhosis declined continuously, especially after the introduction of the direct-acting antiviral agents (DAAs) in 2013. In contrast, alcoholic cirrhosis gained continuously on incidence and has become the second most common underlying disease that led to liver transplantation since 2014. The number of liver transplants for HCC also increased steadily from 13.3% during 1998–2001 to 28.8% during 2010–2013, but declined slightly to 26.6% after 2014 (Figure 1A). Recipient age and donor age increased significantly during 1998–2017. There were significantly more 60–69-year-old recipients during 2014–2017 than during 1998–2001 (33.4 vs. 20.4%, *P* < 0.001), and the fraction of septuagenarian donors was with 25.2% highest during 2014–2017 (Figures 1C,D).

### CIT and Outcome After Liver Transplantation

**Figure 2:**
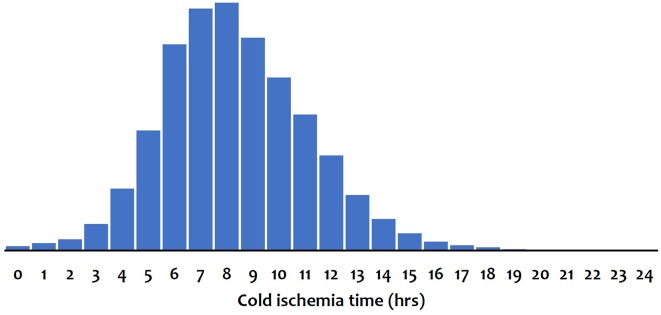
Distribution of cold ischemia time in deceased donor liver transplantations that were performed during 1998–2017 in adult patients.

Over the study period, we observed a shift toward lower CIT. The fraction of transplant cases with ischemia time exceeding 12 h dropped dramatically from 21.5% during 1998–2001 to 6.5% during 2014–2017, and 6–9 h became the most prevalent CIT (Figure 1B). Figure 2 illustrates the distribution of CIT in deceased donor liver transplantations in adult recipients that were performed during 1998–2017 and reported to the CTS. The arithmetic average of the CIT was 8.4 ± 3.0, the median 8, and the inter-quartile range 6–10 h.

**Figure 3:**
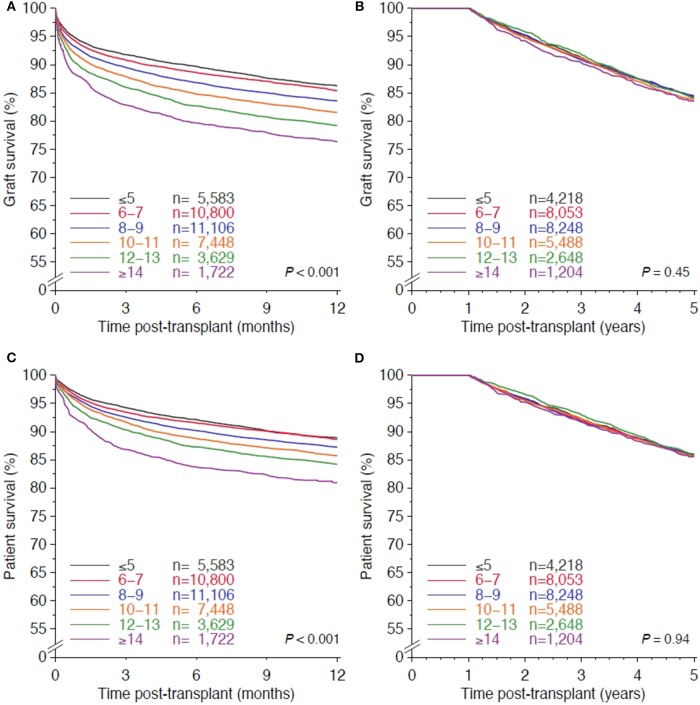
Influence of cold ischemia time on overall graft survival **(A,B)** and patient survival **(C,D)** during first post-transplant year **(A,C)** and after first post-transplant year **(B,D)**. *P*-values of log rank test with linear trend are shown.

As shown in Table 2, Table S1, CIT ≥8 h reduced graft as well as patient survival significantly during the first post-transplant year, but the impact of cold preservation on survival was uneven among liver transplant recipients with different underlying diseases. Overall, graft and patient survival rates declined in a linear fashion as CIT increased (all *P* < 0.001; Figures 3A,C). The multivariable Cox regression analysis indicated a linear influence of CIT, and with each hour added to cold ischemia, the risk of graft loss during the first post-transplant year increased by 3.4% (HR 1.034, 95% CI 1.027–1.041, *P* < 0.001). Remarkably, after the first post-transplant year, CIT did not show a significant effect in the univariate Kaplan-Meier analysis, neither on graft nor on patient survival (*P* = 0.45 and 0.94, respectively; Figures 3B,D).

**Table 2:** Results of the multivariable Cox regression analysis for the influence of CIT on 1-year graft survival in liver transplant recipients with different underlying diseases.

**Cold ischemia time (hours)**	***n***	**HR**	**95 % CI**	***P***
**All underlying diseases**
≤ 5	5,583	1 (ref)	–	–
6–7	10,800	1.04	0.95–1.13	0.40
8–9	11,106	1.14	1.05–1.25	0.003
10–11	7,448	1.22	1.11–1.34	<0.001
12–13	3,629	1.43	1.29–1.59	<0.001
≥14	1,722	1.67	1.47–1.89	<0.001
**HCC**
≤ 5	1,576	1 (ref)	–	–
6–7	3,319	0.92	0.78–1.09	0.36
8–9	3,086	1.06	0.90–1.26	0.47
10–11	1,851	1.33	1.11–1.58	0.002
12–13	798	1.41	1.15–1.74	0.001
≥14	323	1.80	1.39–2.33	<0.001
**HCV-induced liver cirrhosis**
≤ 5	1,341	1 (ref)	–	–
6–7	2,481	1.03	0.87–1.22	0.73
8–9	2,617	1.24	1.05–1.47	0.011
10–11	1,772	1.31	1.10–1.57	0.002
12–13	873	1.51	1.23–1.85	<0.001
≥14	485	1.64	1.30–2.09	<0.001
**Alcoholic cirrhosis**
≤ 5	1,038	1 (ref)	–	–
6–7	1,985	1.14	0.93–1.40	0.21
8–9	2,198	1.07	0.87–1.32	0.50
10–11	1,497	1.09	0.88–1.36	0.42
12–13	779	1.45	1.13–1.84	0.003
≥14	381	1.73	1.31–2.28	<0.001
**Other**
≤ 5	1,628	1 (ref)	–	–
6–7	3,015	1.12	0.94–1.32	0.20
8–9	3,205	1.18	1.00–1.39	0.055
10–11	2,328	1.15	0.96–1.37	0.12
12–13	1,179	1.36	1.11–1.65	0.003
≥14	533	1.59	1.25–2.01	<0.001

*Hazard ratios (HR) with 95% confidence interval (CI) of categorized CIT are shown. HCC, hepatocellular carcinoma; HCV, hepatitis C virus; ref, reference*.

**Figure 4:**
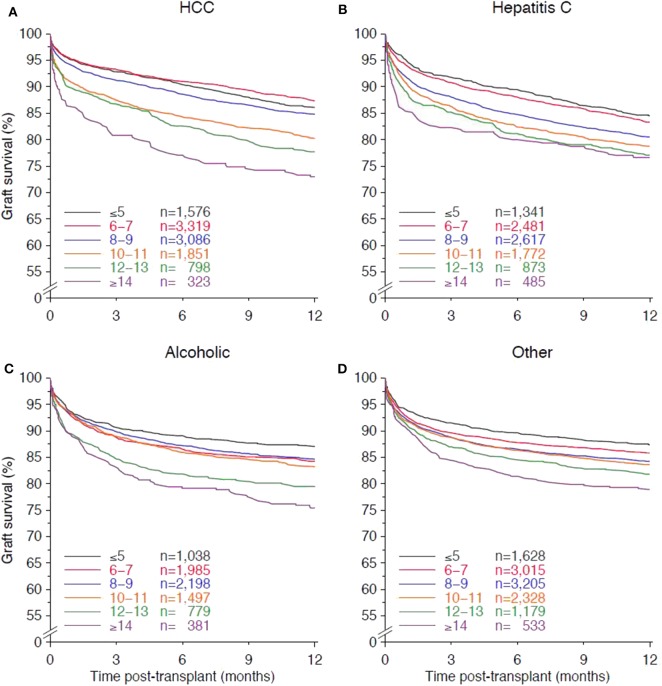
Kaplan-Meier curves demonstrating the impact of cold ischemia time on 1-year graft survival for the main underlying disease categories **(A)** hepatocellular carcinoma (HCC), **(B)** hepatitis C, **(C)** alcoholic cirrhosis, and **(D)** the other less frequent original diseases (autoimmune disorders, cryptogenic cirrhosis, congenital diseases, hepatitis B, metabolic disorders, primary biliary cirrhosis, and primary sclerosing cholangitis). All log rank *P*-values with trend <0.001.

The multivariable Cox regression analysis of the interactions of CIT with other confounders showed a significant interaction only with underlying disease. Prolonged cold ischemia exposed grafts at the highest risk of failure in patients with HCV-cirrhosis. Compared to the reference of <5 h, 8–9 h cold ischemia increased in HCV patients the risk of graft loss by 24% (HR 1.24, 95% CI 1.05–1.47, *P* = 0.011) and ≥14 h cold ischemia by as high as 64% (HR 1.64, 95% CI 1.30–2.09, *P* < 0.001; Table 2). In contrast, grafts transplanted into patients with HCC tolerated longer ischemia times and were at a significantly increased risk of graft loss only if the CIT was 10–11 h (HR 1.33, 95% CI 1.11–1.58, *P* = 0.002) or higher. Most resilient to the negative effect of CIT were grafts transplanted into recipients with cirrhosis due to chronic alcoholism and other underlying diseases (CIT 12–13 h, HR 1.45, 95% CI 1.13–1.84, *P* = 0.003; HR 1.36, 95% CI 1.11–1.65, *P* = 0.003, respectively; Table 2). Similar hazard ratios were obtained in the analysis of patient survival with the exception of HCC patients in whom the mortality risk was in all CIT categories constantly lower than the risk for graft loss (Table S1). Other than in recipients with HCV cirrhosis and other less frequent underlying diseases, 1-year graft survival decreased in a non-linear fashion in recipients with pre-transplant HCC and alcoholic cirrhosis (all *P* < 0.001; Figure 4).

### Underlying Disease and Outcome After Transplantation

**Figure 5:**
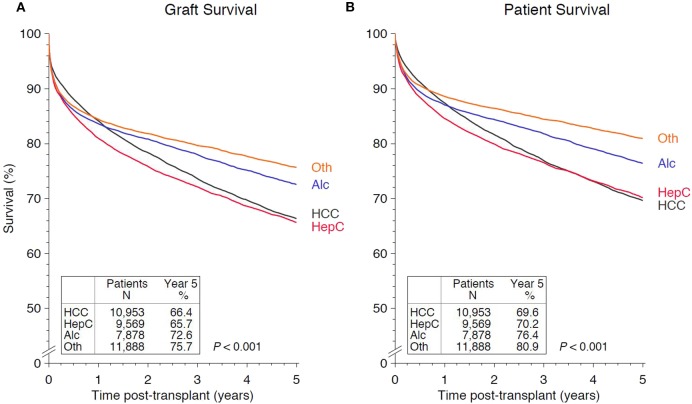
Kaplan-Meier curves demonstrating the impact on **(A)** 5-year graft survival and **(B)** 5-year patient survival of the main original disease categories hepatocellular carcinoma (HCC), hepatitis C (HepC), alcoholic cirrhosis (Alc), and the other less frequent original diseases (autoimmune disorders, cryptogenic cirrhosis, congenital diseases, hepatitis B, metabolic disorders, primary biliary cirrhosis, and primary sclerosing cholangitis) (Oth).

When the three most common underlying diseases were analyzed, grafts transplanted into patients with alcohol-induced liver cirrhosis showed the best (72.6%) and grafts transplanted into recipients with HCV-cirrhosis the worst 5-year survival (65.7%; *P* < 0.001; Figure 5A). Kaplan-Meier curves for patient survival had a similar trend (*P* < 0.001; Figure 5B). Patients with pretransplant HCC demonstrated the best 1-year graft and patient survival, but this worsened in time and declined at year 5 to a rate of 66.4 and 69.6%, respectively (Figures 5A,B).

**Figure 6:**
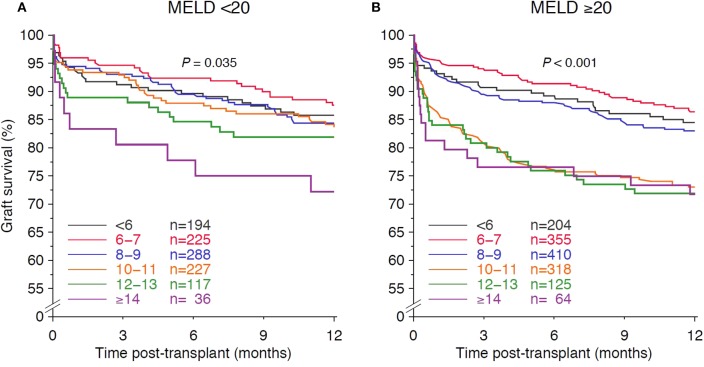
Influence of cold ischemia time on 1-year graft survival in subpopulations of patients transplanted because of hepatocellular carcinoma with **(A)** low and **(B)** high Model of End Stage Liver Disease (MELD) score. Log rank *P*-values with trend are shown.

We analyzed the HCC subpopulation separately for interactions between CIT and confounders. Despite the seemingly large differences in hazard risk ratios of the multivariable Cox regression analysis, no significant interactions were observed, and transplant period, recipient sex, and recipient and donor age did not influence the effect of ≥10 h CIT on graft survival substantially (Table 3). Kaplan-Meier estimation of 1-year graft survival of HCC patients with respect to MELD score categories is shown in Figure 6. CIT ≥10 h worsened 1-year graft survival significantly in HCC recipients with a high MELD score of ≥20, whereas its influence on outcome was less pronounced in patients with a low MELD score of <20 (*P* < 0.001 and 0.035, respectively). In the multivariable Cox regression analysis, the risk of graft loss due to CIT ≥10 h was significantly increased in patients with a MELD score of ≥20 (HR=1.71, 95% CI 1.33–2.21, *P* < 0.001), whereas HCC patients with a MELD score of <20 showed a similar risk only after CIT ≥14 h (HR = 1.67, 95% CI 0.86–3.26, *P* = 0.13).

**Table 3:** Results of multivariable Cox regression analysis for the impact of CIT ≥10 h on 1-year graft survival in subpopulations of patients transplanted because of HCC.

**Subpopulation**	***n***	**Regression coefficient**	**HR**	**95% CI**	***P***
All patients with HCC	10,953	0.335	1.40	1.26–1.55	<0.001
Transplant year					
1998–2007	2,956	0.336	1.40	1.17–1.68	<0.001
2008–2017	7,997	0.336	1.40	1.23–1.59	<0.001
Recipient sex					
Female	1,853	0.139	1.15	0.89–1.49	0.29
Male	9,028	0.364	1.44	1.28–1.62	<0.001
Recipient age (years)					
<65	9,420	0.347	1.42	1.26–1.59	<0.001
≥65	1,533	0.296	1.34	1.04–1.74	0.026
Donor age (years)					
<65	7,328	0.322	1.38	1.21–1.57	<0.001
≥65	3,625	0.371	1.45	1.21–1.74	<0.001

*Regression coefficients, hazard ratios (HR) with 95% confidence interval (CI) of CIT ≥10 h are shown. HCC, hepatocellular carcinoma*.

## Discussion

After prolonged cold ischemia, outcome of a graft depends on its ability to recover from the ischemia injury, which appears to be especially difficult in steatotic grafts or grafts from older donors (12–14). CIT influenced graft and patient survival in a linear fashion and only during the first year after transplantation. At later time points the effect of equidistant 1-h CIT intervals on graft and patient survival was no longer present, indicating that ischemia-reperfusion injury is relevant only during the early post-transplant phase and that if and once the liver has recovered from the influences of ischemia—its duration becomes irrelevant. This effect contrasts with the influence of donor age which has an impact on graft survival also at later time points (8).

CIT is a factor that occurs during allocation and can only be calculated retrospectively. Along with macrovesicular steatosis of >40% and donor age of >65 years, CIT >14 h is a maEDC (2). Prolonged cold ischemia increases the risk of graft failure and early HCC recurrence (2, 6, 7). In our study it affected graft survival in the most common indication groups and is therefore relevant for the organ allocation. However, its negative effects were unevenly distributed among recipients with different indications for liver transplantation. Increasing CIT had a dramatic impact on outcome in HCV recipients. Similar effect of donor age on outcome in HCV recipients has been reported (8, 15). Grafts transplanted into HCV patients appeared to have the lowest tolerance for cold ischemia and were already at a significantly increased risk of graft loss at CIT as low as 8 h. The mechanisms that determine the association between worse outcomes in HCV patients and longer CIT are multifactorial. Together with advanced donor age and macrovesicular steatosis, CIT, as the third maEDC, is an independent risk factor associated with preservation injury, delayed graft function, and biliary complications (2, 5). Preservation injury during cold storage affects post-transplant outcomes strongly, especially in HCV recipients because HCV patients with biopsy-proven preservation injury have been shown to have worse outcomes than HCV recipients without histologically proven injury (16, 17). The preservation injury that follows tissue inflammation, cellular edema, cholestasis, and progressive centrilobular necrosis increases the risk of rejection and biliary complications. After preservation injury and during the regenerative hepatocyte proliferation that follows cellular death, HCV could more effectively infiltrate into the proliferating cells, leading to early aggressive HCV recurrence (16–18). Moreover, preexisting illnesses, malnutrition, cytomegalovirus infection, and HCV-positive donors have been identified as factors that may also contribute to HCV recurrence after liver transplantation. However, HCV genotype 1, high viral load, induction immunosuppression before transplantation and overshooting immunosuppression during graft rejection episodes, alone or in combination with advanced donor age and biliary complications, are considered to be the most prominent causes responsible for the increased risk of aggressive HCV recurrence and subsequent graft injury or failure (18, 19). Aggressive immunosuppression regimens rather than the direct effect of a specific immunosuppressive agent might affect the outcome and reducing the intensity of immunosuppression in HCV patients to maintain adequate host immune responses could decrease the HCV recurrence and improve graft and patient survival (20). HCV-related cirrhosis is associated with high rate of recurrence and graft loss, but the introduction of DAAs in 2013 improved graft survival significantly in patients with HCV and modified the course of recurrent HCV-graft disease. HCV slowly but steadily disappears as an indication for liver transplantation, however, DAAs are expensive and not readily available worldwide and specific data on DAAs are not documented in the CTS (8, 16, 18–20). Therefore, it can be assumed that these agents were not comprehensively available for the entire study population.

Although cold ischemia dramatically increased the risk of failure in the HCV subgroup, merely allocating grafts with longer cold ischemia to non-HCV recipients would not sufficiently solve the problem of matching grafts and recipients adequately because grafts transplanted into recipients with alcoholic cirrhosis and patients with HCC were also affected by cold ischemia. Indeed, transplant outcome of patients with alcoholic cirrhosis was influenced by cold ischemia, but these patients were at increased risk of graft loss only after an ischemia time of 12 h. This observation is very interesting and may be attributed to fast recovery once the patient has ceased to consume alcohol. The influence of prolonged ischemia time was also less pronounced in HCC patients compared to the HCV subgroup. HCC patients may have a more suppressed immune state than HCV patients and generate less rigorous immune responses under CIT-mediated inflammation. A recent CTS report by Unterrainer et al. indicated that renal transplant recipients with different forms of pre-transplant cancer had a generally decreased risk of death-censored graft loss, which approximates the rate of immunological graft failures (21). This finding supported the assumption that the patients' deficient immunological surveillance against tumors was paralleled by a weakness in mounting rigorous immunological rejection against the transplant. This may also be true for patients with HCC in whom, due to a generally suppressed immune state, CIT-mediated ischemia-reperfusion injury results in a less rigorous inflammation and rejection. In contrast, CIT can cause a more rigorous inflammation and damage in HCV patients due to an immune environment that is strongly activated by HCV infection. HCC patients received most of the elderly grafts but with the shortest cold ischemia. Allocation of grafts from older donors to recipients with HCC can well be justified because they show the lowest rise in the donor age-dependent risk of graft loss (8). This may explain why HCC patients had the best 1-year graft and patient survival despite the negative influence of CIT with an obvious 10-h cutoff. Graft and patient survival of HCC patients worsened at later time points and were nearly similarly as low as in HCV-patients, but this may also be attributed to recurrence of HCC that led to death of the patient with functioning graft. This assumption could not be definitely verified, as death with functioning graft could not be reliably examined in this multicenter study. CIT had different effect on graft survival in patients with HCC and different MELD scores. While HCC patients with a MELD score of <20 tolerated cold ischemia of up to <14 h, more than 25% of the grafts with cold ischemia longer than 10 h succumbed to failure if the recipient had a MELD score of ≥20, which is an extremely poor outcome considering the current 1-year graft survival benchmarks in patients with HCC (9, 10). This clearly suggests that with the increase of the MELD score, the tolerance of prolonged cold ischemia decreases. Because allocating grafts with longer CIT to the aforementioned recipient category did not carry disproportionate risk, this type of matching (longer CIT with HCC recipients and MELD <20) is in line with previous findings and may be acceptable when facing organ shortage (2, 5). Patient survival constantly better than graft survival, also after longer CIT, was observed only in recipients with HCC. This may be attributed to the higher resilience of a re-transplant in patients with HCC. While Goldaracena et al. showed that patients with high labMELD scores benefit from transplantation as soon as possible and irrespective of the organ quality, our two recent studies pointed out that exact match between graft and recipient is important, and that grafts with maEDC could be allocated to low-risk patients with labMELD <20 e.g., patients with HCC (2, 5, 22). These findings were confirmed in a recent large cohort CTS study (8). Discrepant results may be attributed to the lack of uniform donor-recipient matching, but the aforementioned studies and the results of the current study indicate that matched allocation is plausible. However, the results of CIT with ≥14-h cutoff should be interpreted with great caution because cold ischemia exceeded 13 h only in 2.9% of HCC patients and in 4.3% of all recipients. Moreover, regarding the MELD score as a single surrogate parameter for the patient's condition bears a risk of bias. Also, for the purpose of this study, MELD score was only partially available since 2007 and has the known disadvantage of potentially inconsistent data entries due to the commingling of laboratory and exceptional MELD score values. Hence, the interaction of cold ischemia and MELD score demands further clarification. Nevertheless, to reduce the risk for individual patients, avoiding unfavorable constellations, e.g., HCV-patients and grafts with long cold preservation time, is prudent. The relevance of HCV-associated cirrhosis is decreasing owing to improved DAAs therapy and the new challenge is how to choose the most suitable candidate for grafts with longer cold ischemia out of recipients with HCC, alcoholic cirrhosis, and other diseases that gain on significance (23, 24). Organs with longer cold ischemia may be preferred for non-HCV recipients e.g., patients with HCC or alcoholic liver cirrhosis, but such ischemia time limits may only be useful in recipients with MELD scores below 20 as they do not impair outcome in this subgroup.

DRI and ET-DRI calculations include donor age, cause of death, donation after cardiac death, partial or split liver, location, and CIT (25, 26). With the exception of “location,” all of the aforementioned risk factors were considered in our Cox regression model. Location was expected to play a less important role in our predominantly European cohort. We therefore took this parameter indirectly into account and used the confounder CIT instead. In line with the findings of Feng et al., we found the influence of CIT to be linear and similarly strong (HR_DRI_ = e^0.010^= 1.010; HR_CTS_ = e^0.034^= 1.034). However, our result showed that cold ischemia is important only during the first year following transplantation, and that its influence depends on the indication for transplantation. The studies of 20,023 recipients by Feng et al., and of 6,621 recipients by Braat et al. analyzed the effect on total available follow-up (DRI median 3 years; ET-DRI median 2.5 years), assumed constant linear influence of CIT, and did not consider indication for transplantation as confounder. Since the influence of ischemia time is clearly greatest at the beginning, the regression coefficient diminishes with the increase of the follow-up time, which is why we found it to be 3.4 times higher in our data than the coefficient used for the calculation of the DRI and ET-DRI (25, 26). According to the DRI- and ET-DRI calculations, CIT has an assumed linear influence on the outcome after transplantation (coefficient used for cold ischemia is 0.010) (25, 26). The linearity of cold ischemia can be assumed when the increase of ischemia time per hour would always affect the graft survival in a similar manner independent of the range of the ischemia time. By assuming the linear influence and by setting a fixed coefficient as in the aforementioned formulas, the categorical effect of CIT cannot be observed, especially when there is evidence of the opposite, non-linear influence. Not being able to retrieve DRI from the CTS database limits our study. However, the influence of cold ischemia is clustered in a non-linear fashion in recipients with HCC and alcoholic liver cirrhosis, and several cutoffs stand out. In HCC recipients, CIT only makes a difference when the comparison is made between ischemia time <10 h and ≥10 h, whereas in patients with alcoholic cirrhosis the two cutoffs are at 10 and 12 h. Therefore, similar to donor age, a categorical model that also considers the underlying disease should be preferred to a linear one in the case of CIT (8). The awareness of the importance of cold ischemia has increased significantly over the years, and the formulas for the calculation of DRI and ET-DRI are based on data from 2002 to 2007, respectively. Therefore, entering the indication for transplantation and CIT as categorical variable for HCC and for alcoholic cirrhosis with 3 different categories (HCC: <10 h, 10–13 h, and ≥14 h; alcoholic cirrhosis: <12 h, ≥12 h), and their respective coefficients may be worth considering as it might increase the specificity of the DRIs.

The allocation process is complex, but CIT can be managed by improved internal organization and regional allocation if estimated cold ischemia exceeds certain limits (2, 27). Our study of more than 40,000 patients revealed a strong negative linear impact of CIT on 1-year graft and patient survival. Remarkably, the negative influence of different CIT vanished after the first year suggesting that other factors come into play. We narrowed the parameters that did not contribute substantially to the negative effect of longer cold ischemia to recipient gender and age ≥65 years, and HCC patients with a MELD score of <20. The negative cold ischemia effect depends strongly on the underlying disease. While HCC patients and recipients with alcoholic cirrhosis are able to compensate better for the effect of longer CIT, the impact of CIT is most severe in patients with HCV-related cirrhosis and should not exceed 8 h. Optimal donor-recipient matching is crucial in achieving reasonable outcomes after transplantation, and taking underlying disease into consideration is important especially in allocation of maEDC organs.

## Data Availability Statement

The raw data are available to the Collaborative Transplant Study in accordance with the consents of the patients, the participating transplant centers and registries.

## Ethics Statement

The work of the CTS is approved by the Ethics Committee of the Medical Faculty of Heidelberg University (No. 083/2005) and performed in accordance with the World Medical Association Declaration of Helsinki Ethical Principles in the currently valid version.

## Author Contributions

VL designed the study, analyzed data, and wrote the manuscript. BD participated in writing of the manuscript, performance of research, and data analysis. KW participated in data analysis. AM designed the study and analyzed data. CS designed the study, analyzed data, and participated in writing of the manuscript.

### Conflict of Interest

The authors declare that the research was conducted in the absence of any commercial or financial relationships that could be construed as a potential conflict of interest.

## Abbreviations

CI: confidence interval
CIT: Cold Ischemia Time
CTS: Collaborative Transplant Study
DAAs: Direct-acting Antiviral Agents
DRI: Donor Risk Index
EDC: Extended Donor Criteria
ET: Eurotransplant
ET-DRI: Eurotransplant Donor Risk Index
HCC: hepatocellular carcinoma
HCV, Hepatitis C Virus, HR: hazard ratio
IQR: interquartile range
labMELD: laboratory Model of End Stage Liver Disease
maEDC: major Extended Donor Criteria.
