# Supplementary material for: The Differential Influence of Cold Ischemia Time on Outcome After Liver Transplantation for Different Indications—Who Is at Risk? A Collaborative Transplant Study Report
Source: Front Immunol. 2020 May 12;11:892. doi: 10.3389/fimmu.2020.00892 (PMC7235423; doi:10.3389/fimmu.2020.00892)
Supplement: Supplementary file 1 [file Data_Sheet_1.PDF]

Supplemental Material

Table S1. Results of the multivariable Cox regression analysis for the influence of cold ischemia time (CIT) on patient survival at year 1 in liver transplant recipients with different underlying diseases. Hazard ratios (HR) with 95% confidence interval (CI) of categorized CIT are shown.

Cold ischemia time (hours)	n	HR	95 % CI	*P*
**All underlying diseases**				
≤5	5,583	1 (ref)	–	–
6–7	10,800	1.01	0.92–1.12	0.77
8–9	11,106	1.12	1.01–1.23	0.029
10–11	7,448	1.18	1.07–1.31	0.001
12–13	3,629	1.36	1.21–1.53	<0.001
≥14	1,722	1.70	1.48–1.95	<0.001
**HCC**				
≤5	1,576	1 (ref)	–	–
6–7	3,319	0.89	0.74–1.07	0.22
8–9	3,086	1.01	0.84–1.22	0.89
10–11	1,851	1.26	1.04–1.53	0.020
12–13	798	1.35	1.07–1.70	0.011
≥14	323	1.58	1.18–2.11	0.002
**HCV-induced liver cirrhosis**				
≤5	1,341	1 (ref)	–	–
6–7	2,481	1.01	0.84–1.22	0.89
8–9	2,617	1.23	1.03–1.48	0.024
10–11	1,772	1.24	1.02–1.50	0.033
12–13	873	1.44	1.15–1.81	0.001
≥14	485	1.60	1.23–2.09	<0.001
**Alcoholic cirrhosis**				
≤5	1,038	1 (ref)	–	–
6–7	1,985	1.10	0.87–1.38	0.44
8–9	2,198	1.05	0.83–1.32	0.70
10–11	1,497	1.09	0.85–1.39	0.51
12–13	779	1.35	1.03–1.78	0.031
≥14	381	1.79	1.32–2.43	<0.001
**Other**				
≤5	1,628	1 (ref)	–	–
6–7	3,015	1.13	0.93–1.38	0.23
8–9	3,205	1.16	0.96–1.42	0.13
10–11	2,328	1.16	0.94–1.42	0.17
12–13	1,179	1.29	1.02–1.63	0.033
≥14	533	1.83	1.40–2.39	<0.001

HCC, hepatocellular carcinoma; HCV, hepatitis C virus; ref, reference
